# Dicyclo­hexyl­ammonium thio­cyanate: monoclinic polymorph

**DOI:** 10.1107/S1600536811040001

**Published:** 2011-10-05

**Authors:** N. Selvakumaran, R. Karvembu, Seik Weng Ng, Edward R. T. Tiekink

**Affiliations:** a Department of Chemistry, National Institute of Technology, Tiruchirappalli 620 015, India; b Department of Chemistry, University of Malaya, 50603 Kuala Lumpur, Malaysia; c Chemistry Department, Faculty of Science, King Abdulaziz University, PO Box 80203 Jeddah, Saudi Arabia

## Abstract

The title salt, C_12_H_24_N^+^·NCS^−^, represents a monoclinic polymorph of the previously reported ortho­rhom­bic form [Khawar Rauf *et al.* (2008 ▶). *Acta Cryst*. E**64**, o366]. Two independent formula units comprise the asymmetric unit with the major difference in their mol­ecular structures relating to the relative dispositions of the cyclo­hexyl rings [dihedral angles = 79.88 (6) and 67.72 (5)°]. Further, the independent anions form distinctive patterns of hydrogen-bonding inter­actions, *i.e*. 2 × N—H⋯N *versus* N—H⋯N and N—H⋯S. The resulting supra­molecular architecture is a supra­molecular chain along the *c* axis based on a square-wave topology.

## Related literature

For the crystal structure of the ortho­rhom­bic polymorph, see: Khawar Rauf *et al.* (2008 ▶). For additional structure analysis, see: Spek (2009 ▶).
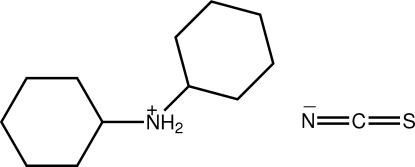


## Experimental

### 

#### Crystal data

C_12_H_24_N^+^·NCS^−^
*M* *_r_* = 240.40
Monoclinic, 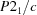

*a* = 8.5190 (1) Å
*b* = 37.9428 (5) Å
*c* = 8.5578 (1) Å
β = 93.661 (1)°
*V* = 2760.53 (6) Å^3^
*Z* = 8
Cu *K*α radiation
μ = 1.88 mm^−1^
*T* = 100 K
0.30 × 0.30 × 0.20 mm

#### Data collection

Agilent SuperNova Dual diffractometer with an Atlas detector
Absorption correction: multi-scan (*CrysAlis PRO*; Agilent, 2010 ▶) *T*_min_ = 0.602, *T*_max_ = 0.704
16974 measured reflections
5693 independent reflections
5363 reflections with *I* > 2σ(*I*)
*R*_int_ = 0.020

#### Refinement

*R*[*F* ^2^ > 2σ(*F* ^2^)] = 0.030
*wR*(*F* ^2^) = 0.079
*S* = 1.02
5693 reflections
305 parameters
H atoms treated by a mixture of independent and constrained refinement
Δρ_max_ = 0.29 e Å^−3^
Δρ_min_ = −0.25 e Å^−3^

### 

Data collection: *CrysAlis PRO* (Agilent, 2010 ▶); cell refinement: *CrysAlis PRO*; data reduction: *CrysAlis PRO*; program(s) used to solve structure: *SHELXS97* (Sheldrick, 2008 ▶); program(s) used to refine structure: *SHELXL97* (Sheldrick, 2008 ▶); molecular graphics: *ORTEP-3* (Farrugia, 1997 ▶), *QMol* (Gans & Shalloway, 2001 ▶) and *DIAMOND* (Brandenburg, 2006 ▶); software used to prepare material for publication: *publCIF* (Westrip, 2010 ▶).

## Supplementary Material

Crystal structure: contains datablock(s) global, I. DOI: 10.1107/S1600536811040001/hg5105sup1.cif

Structure factors: contains datablock(s) I. DOI: 10.1107/S1600536811040001/hg5105Isup2.hkl

Supplementary material file. DOI: 10.1107/S1600536811040001/hg5105Isup3.cml

Additional supplementary materials:  crystallographic information; 3D view; checkCIF report

## Figures and Tables

**Table 1:** Hydrogen-bond geometry (Å, °)

*D*—H⋯*A*	*D*—H	H⋯*A*	*D*⋯*A*	*D*—H⋯*A*
N1—H11⋯N3	0.887 (15)	2.091 (15)	2.9696 (13)	170.6 (13)
N1—H12⋯N4^i^	0.960 (16)	1.900 (16)	2.8539 (13)	172.1 (13)
N2—H21⋯N3	0.911 (15)	2.068 (15)	2.9650 (12)	168.1 (13)
N2—H22⋯S2	0.895 (16)	2.475 (16)	3.3544 (9)	167.4 (13)

Symmetry code: (i) 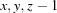.

**Fig. 1.**
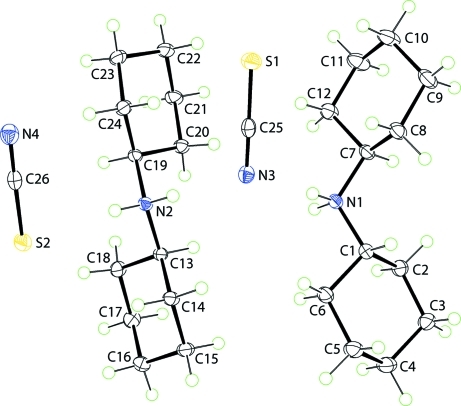
The molecular structures of the ions comprising (I) showing the atom-labelling scheme and displacement ellipsoids at the 50% probability level.

**Fig. 2.**
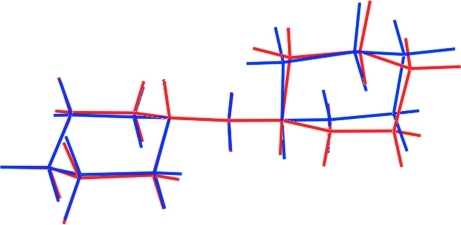
An overlay diagram of the two cations in (I) emphasizing the minor variation in conformation. The red molecule illustrates the N1-containing cation.

**Fig. 3.**
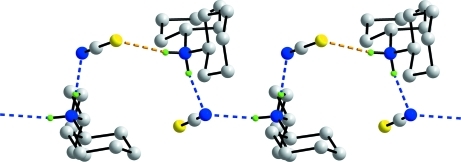
A supramolecular chain in (I) mediated by N—H···N and N—H···S hydrogen bonding shown as blue and orange dashed lines, respectively. Hydrogen atoms not participating hydrogen bonding contacts have been omitted for reasons of clarity.

**Fig. 4.**
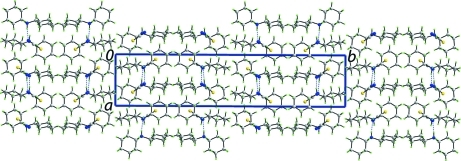
A view in projection down the c axis of the unit-cell contents of (I).

## supplementary crystallographic information

### Comment

The crystal structure of the title salt (I) represents a monoclinic form of the previously reported (at 123 K) orthorhombic form (Khawar Rauf *et al.*, 2008). In the latter, one formula unit comprises the asymmetric unit whereas two independent formula units comprise the asymmetric unit in (I), Fig. 1. The differences in the structure of (I) relate to a minor variation in the orientation of the cyclohexyl groups, Fig. 2, and in the nature of the intermolecular interactions they form, see below. In terms of molecular structure, each cyclohexyl ring has a chair conformation and the r.m.s. differences for the cations are 0.0026 Å for distances and 0.530° for angles (Spek, 2009). The different orientations are probably best described by the dihedral angles formed between the least-squares planes through the pairs of rings, *i.e*. 79.88 (6) and 67.72 (5)°, for the N1- and N2-cations, respectively.

In terms of crystal packing, the N1-cation forms N—H···N hydrogen bonds exclusively whereas the N2-cation forms a N—H···N and a N—H···S hydrogen bond, Table 1. The result of the hydrogen bonding is the formation of a supramolecular chain with a square-wave topology along the *c* axis. The N1-cation bridges two N2-cations *via* N—H···N hydrogen bonds and the N2-cation bridges two N1-cations *via* the N and S atoms, Fig. 3. Chains assemble into layers in the *ac* plane which stack along the *b* axis, Fig. 4. In the orthorhombic polymorph, the thiocyanate anion bridges two cations *via* the N and S atoms to form a supramolecular chain.

### Experimental

The title compound was obtained as an unexpected product from a reaction mixture containing dicyclohexylamine, isopthaloyl dichloride and potassium thiocyanate in acetone under reflux conditions. Crystals were grown from a solution of the compound in ethylacetate / petroleum ether (1:3).

### Refinement

The H-atoms were placed in calculated positions (C—H 0.99 to 1.00 Å) and were included in the refinement in the riding model approximation, with *U*_iso_(H) set to 1.2*U*_equiv_(C). The ammonium-H atoms were refined without restraint.

### Crystal data

**Table:**

C_12_H_24_N^+^·NCS^−^	*F*(000) = 1056
*M**_r_* = 240.40	*D*_x_ = 1.157 Mg m^−^^3^
Monoclinic, *P*2_1_/*c*	Cu *K*α radiation, λ = 1.54184 Å
Hall symbol: -P 2ybc	Cell parameters from 9471 reflections
*a* = 8.5190 (1) Å	θ = 3.5–76.6°
*b* = 37.9428 (5) Å	µ = 1.88 mm^−^^1^
*c* = 8.5578 (1) Å	*T* = 100 K
β = 93.661 (1)°	Prism, colourless
*V* = 2760.53 (6) Å^3^	0.30 × 0.30 × 0.20 mm
*Z* = 8	

### Data collection

**Table:**

Agilent SuperNova Dual diffractometer with an Atlas detector	5693 independent reflections
Radiation source: SuperNova (Cu) X-ray Source	5363 reflections with *I* > 2σ(*I*)
Mirror	*R*_int_ = 0.020
Detector resolution: 10.4041 pixels mm^-1^	θ_max_ = 75.0°, θ_min_ = 5.2°
ω scans	*h* = −9→10
Absorption correction: multi-scan (*CrysAlis PRO*; Agilent, 2010)	*k* = −45→47
*T*_min_ = 0.602, *T*_max_ = 0.704	*l* = −9→10
16974 measured reflections	

### Refinement

**Table:**

Refinement on *F*^2^	Primary atom site location: structure-invariant direct methods
Least-squares matrix: full	Secondary atom site location: difference Fourier map
*R*[*F*^2^ > 2σ(*F*^2^)] = 0.030	Hydrogen site location: inferred from neighbouring sites
*wR*(*F*^2^) = 0.079	H atoms treated by a mixture of independent and constrained refinement
*S* = 1.02	*w* = 1/[σ^2^(*F*_o_^2^) + (0.0402*P*)^2^ + 0.9255*P*] where *P* = (*F*_o_^2^ + 2*F*_c_^2^)/3
5693 reflections	(Δ/σ)_max_ = 0.002
305 parameters	Δρ_max_ = 0.29 e Å^−^^3^
0 restraints	Δρ_min_ = −0.25 e Å^−^^3^

### Special details

**Table:**

Geometry. All e.s.d.'s (except the e.s.d. in the dihedral angle between two l.s. planes) are estimated using the full covariance matrix. The cell e.s.d.'s are taken into account individually in the estimation of e.s.d.'s in distances, angles and torsion angles; correlations between e.s.d.'s in cell parameters are only used when they are defined by crystal symmetry. An approximate (isotropic) treatment of cell e.s.d.'s is used for estimating e.s.d.'s involving l.s. planes.
Refinement. Refinement of *F*^2^ against ALL reflections. The weighted *R*-factor *wR* and goodness of fit *S* are based on *F*^2^, conventional *R*-factors *R* are based on *F*, with *F* set to zero for negative *F*^2^. The threshold expression of *F*^2^ > σ(*F*^2^) is used only for calculating *R*-factors(gt) *etc*. and is not relevant to the choice of reflections for refinement. *R*-factors based on *F*^2^ are statistically about twice as large as those based on *F*, and *R*- factors based on ALL data will be even larger.

### Fractional atomic coordinates and isotropic or equivalent isotropic displacement parameters (Å^2^)

**Table:**

	*x*	*y*	*z*	*U*_iso_*/*U*_eq_	
S1	0.09309 (3)	0.325069 (7)	0.66579 (3)	0.02219 (8)	
S2	0.73345 (3)	0.424830 (7)	0.97466 (3)	0.02176 (8)	
N1	0.24296 (10)	0.37649 (2)	0.18494 (11)	0.01476 (18)	
N2	0.59395 (10)	0.38563 (2)	0.64295 (10)	0.01326 (17)	
N3	0.25785 (10)	0.38031 (2)	0.53216 (10)	0.01739 (19)	
N4	0.57319 (11)	0.37474 (3)	1.14655 (11)	0.0213 (2)	
C1	0.18788 (12)	0.41052 (3)	0.10828 (12)	0.0154 (2)	
H1	0.0728	0.4134	0.1215	0.018*	
C2	0.21502 (14)	0.41008 (3)	−0.06624 (12)	0.0192 (2)	
H2A	0.1487	0.3916	−0.1187	0.023*	
H2B	0.3265	0.4043	−0.0810	0.023*	
C3	0.17461 (15)	0.44596 (3)	−0.14073 (13)	0.0231 (2)	
H3A	0.2005	0.4456	−0.2519	0.028*	
H3B	0.0602	0.4502	−0.1377	0.028*	
C4	0.26455 (16)	0.47591 (3)	−0.05596 (14)	0.0251 (2)	
H4A	0.3787	0.4730	−0.0673	0.030*	
H4B	0.2319	0.4987	−0.1038	0.030*	
C5	0.23223 (15)	0.47606 (3)	0.11732 (13)	0.0233 (2)	
H5A	0.2940	0.4951	0.1714	0.028*	
H5B	0.1193	0.4809	0.1288	0.028*	
C6	0.27613 (13)	0.44066 (3)	0.19307 (12)	0.0178 (2)	
H6A	0.2502	0.4410	0.3042	0.021*	
H6B	0.3908	0.4368	0.1898	0.021*	
C7	0.16086 (12)	0.34292 (3)	0.13194 (12)	0.0166 (2)	
H7	0.1640	0.3409	0.0157	0.020*	
C8	−0.01021 (13)	0.34341 (3)	0.17372 (14)	0.0212 (2)	
H8A	−0.0661	0.3632	0.1189	0.025*	
H8B	−0.0153	0.3470	0.2878	0.025*	
C9	−0.08997 (14)	0.30851 (3)	0.12608 (16)	0.0269 (3)	
H9A	−0.1996	0.3087	0.1581	0.032*	
H9B	−0.0933	0.3060	0.0108	0.032*	
C10	−0.00232 (15)	0.27727 (3)	0.20182 (17)	0.0279 (3)	
H10A	−0.0537	0.2551	0.1655	0.033*	
H10B	−0.0072	0.2786	0.3169	0.033*	
C11	0.16942 (15)	0.27704 (3)	0.16025 (16)	0.0275 (3)	
H11A	0.1745	0.2734	0.0461	0.033*	
H11B	0.2253	0.2572	0.2147	0.033*	
C12	0.25028 (13)	0.31181 (3)	0.20785 (14)	0.0205 (2)	
H12A	0.2544	0.3143	0.3232	0.025*	
H12B	0.3595	0.3117	0.1748	0.025*	
C13	0.66417 (12)	0.40923 (3)	0.52378 (12)	0.0133 (2)	
H13	0.6382	0.3993	0.4170	0.016*	
C14	0.58766 (12)	0.44545 (3)	0.53387 (12)	0.0156 (2)	
H14A	0.6065	0.4550	0.6411	0.019*	
H14B	0.4726	0.4434	0.5110	0.019*	
C15	0.65691 (13)	0.47051 (3)	0.41591 (13)	0.0184 (2)	
H15A	0.6320	0.4617	0.3083	0.022*	
H15B	0.6089	0.4941	0.4245	0.022*	
C16	0.83523 (13)	0.47332 (3)	0.44655 (13)	0.0198 (2)	
H16A	0.8785	0.4891	0.3678	0.024*	
H16B	0.8600	0.4836	0.5515	0.024*	
C17	0.91103 (13)	0.43707 (3)	0.43778 (13)	0.0197 (2)	
H17A	1.0257	0.4392	0.4625	0.024*	
H17B	0.8946	0.4279	0.3297	0.024*	
C18	0.84242 (12)	0.41101 (3)	0.55173 (13)	0.0173 (2)	
H18A	0.8881	0.3873	0.5370	0.021*	
H18B	0.8701	0.4186	0.6607	0.021*	
C19	0.64807 (12)	0.34769 (3)	0.65169 (12)	0.0141 (2)	
H19	0.7622	0.3470	0.6872	0.017*	
C20	0.62609 (13)	0.33067 (3)	0.49062 (12)	0.0164 (2)	
H20A	0.6962	0.3422	0.4182	0.020*	
H20B	0.5161	0.3340	0.4485	0.020*	
C21	0.66374 (14)	0.29126 (3)	0.50033 (13)	0.0200 (2)	
H21A	0.6432	0.2804	0.3958	0.024*	
H21B	0.7767	0.2881	0.5319	0.024*	
C22	0.56472 (14)	0.27271 (3)	0.61790 (13)	0.0215 (2)	
H22A	0.4521	0.2742	0.5821	0.026*	
H22B	0.5941	0.2475	0.6244	0.026*	
C23	0.59016 (14)	0.28959 (3)	0.77911 (13)	0.0207 (2)	
H23A	0.7007	0.2860	0.8189	0.025*	
H23B	0.5214	0.2780	0.8526	0.025*	
C24	0.55370 (13)	0.32905 (3)	0.77289 (12)	0.0176 (2)	
H24A	0.4400	0.3325	0.7462	0.021*	
H24B	0.5791	0.3396	0.8773	0.021*	
C25	0.18777 (12)	0.35743 (3)	0.58834 (12)	0.0165 (2)	
C26	0.64060 (12)	0.39560 (3)	1.07589 (12)	0.0161 (2)	
H11	0.2359 (16)	0.3784 (4)	0.2875 (18)	0.021 (3)*	
H12	0.3529 (19)	0.3737 (4)	0.1698 (17)	0.028 (4)*	
H21	0.4878 (18)	0.3857 (4)	0.6218 (16)	0.021 (3)*	
H22	0.6162 (18)	0.3952 (4)	0.7374 (18)	0.026 (4)*	

### Atomic displacement parameters (Å^2^)

**Table:**

	*U*^11^	*U*^22^	*U*^33^	*U*^12^	*U*^13^	*U*^23^
S1	0.01980 (14)	0.01931 (14)	0.02743 (15)	−0.00320 (10)	0.00126 (11)	0.00326 (10)
S2	0.02543 (15)	0.02238 (14)	0.01734 (14)	−0.00632 (10)	0.00034 (10)	0.00057 (10)
N1	0.0141 (4)	0.0151 (4)	0.0149 (4)	−0.0022 (3)	0.0002 (3)	−0.0004 (3)
N2	0.0136 (4)	0.0112 (4)	0.0149 (4)	0.0000 (3)	0.0004 (3)	−0.0006 (3)
N3	0.0156 (4)	0.0193 (4)	0.0171 (4)	0.0009 (3)	−0.0003 (3)	0.0006 (3)
N4	0.0176 (4)	0.0264 (5)	0.0197 (5)	−0.0008 (4)	0.0010 (4)	0.0001 (4)
C1	0.0149 (5)	0.0152 (5)	0.0158 (5)	−0.0008 (4)	0.0000 (4)	−0.0001 (4)
C2	0.0250 (5)	0.0176 (5)	0.0149 (5)	−0.0016 (4)	−0.0008 (4)	−0.0009 (4)
C3	0.0318 (6)	0.0196 (5)	0.0173 (5)	−0.0018 (5)	−0.0031 (5)	0.0022 (4)
C4	0.0354 (7)	0.0179 (5)	0.0218 (6)	−0.0054 (5)	−0.0003 (5)	0.0033 (4)
C5	0.0318 (6)	0.0155 (5)	0.0224 (6)	−0.0023 (4)	−0.0001 (5)	−0.0018 (4)
C6	0.0203 (5)	0.0165 (5)	0.0164 (5)	−0.0028 (4)	0.0001 (4)	−0.0022 (4)
C7	0.0168 (5)	0.0153 (5)	0.0175 (5)	−0.0037 (4)	−0.0001 (4)	−0.0018 (4)
C8	0.0144 (5)	0.0176 (5)	0.0312 (6)	−0.0019 (4)	−0.0018 (4)	0.0001 (4)
C9	0.0189 (5)	0.0239 (6)	0.0374 (7)	−0.0073 (5)	−0.0032 (5)	−0.0024 (5)
C10	0.0249 (6)	0.0164 (5)	0.0425 (7)	−0.0068 (5)	0.0036 (5)	−0.0021 (5)
C11	0.0256 (6)	0.0160 (5)	0.0414 (7)	−0.0015 (5)	0.0058 (5)	−0.0056 (5)
C12	0.0162 (5)	0.0165 (5)	0.0288 (6)	0.0001 (4)	0.0016 (4)	−0.0008 (4)
C13	0.0142 (5)	0.0119 (5)	0.0137 (5)	−0.0009 (4)	0.0013 (4)	0.0003 (4)
C14	0.0144 (5)	0.0122 (5)	0.0204 (5)	−0.0002 (4)	0.0013 (4)	0.0005 (4)
C15	0.0172 (5)	0.0141 (5)	0.0238 (5)	−0.0007 (4)	0.0005 (4)	0.0041 (4)
C16	0.0181 (5)	0.0187 (5)	0.0225 (5)	−0.0059 (4)	0.0008 (4)	0.0026 (4)
C17	0.0138 (5)	0.0241 (6)	0.0215 (5)	−0.0010 (4)	0.0034 (4)	0.0029 (4)
C18	0.0136 (5)	0.0177 (5)	0.0205 (5)	0.0013 (4)	0.0012 (4)	0.0021 (4)
C19	0.0144 (5)	0.0098 (5)	0.0176 (5)	0.0007 (4)	−0.0019 (4)	0.0000 (4)
C20	0.0206 (5)	0.0130 (5)	0.0158 (5)	−0.0007 (4)	0.0017 (4)	−0.0005 (4)
C21	0.0265 (6)	0.0122 (5)	0.0214 (5)	−0.0003 (4)	0.0024 (4)	−0.0029 (4)
C22	0.0304 (6)	0.0127 (5)	0.0211 (5)	−0.0034 (4)	−0.0009 (5)	0.0002 (4)
C23	0.0300 (6)	0.0137 (5)	0.0181 (5)	−0.0001 (4)	−0.0019 (4)	0.0029 (4)
C24	0.0231 (5)	0.0146 (5)	0.0149 (5)	0.0004 (4)	0.0004 (4)	0.0012 (4)
C25	0.0133 (5)	0.0196 (5)	0.0161 (5)	0.0035 (4)	−0.0021 (4)	−0.0024 (4)
C26	0.0132 (5)	0.0207 (5)	0.0142 (5)	0.0026 (4)	−0.0015 (4)	−0.0040 (4)

### Geometric parameters (Å, °)

**Table:**

S1—C25	1.6327 (11)	C10—H10B	0.9900
S2—C26	1.6412 (11)	C11—C12	1.5314 (15)
N1—C7	1.5087 (13)	C11—H11A	0.9900
N1—C1	1.5097 (13)	C11—H11B	0.9900
N1—H11	0.887 (15)	C12—H12A	0.9900
N1—H12	0.960 (16)	C12—H12B	0.9900
N2—C13	1.5090 (13)	C13—C18	1.5236 (14)
N2—C19	1.5120 (12)	C13—C14	1.5260 (13)
N2—H21	0.911 (15)	C13—H13	1.0000
N2—H22	0.895 (16)	C14—C15	1.5319 (14)
N3—C25	1.1738 (15)	C14—H14A	0.9900
N4—C26	1.1690 (15)	C14—H14B	0.9900
C1—C2	1.5259 (14)	C15—C16	1.5287 (15)
C1—C6	1.5264 (14)	C15—H15A	0.9900
C1—H1	1.0000	C15—H15B	0.9900
C2—C3	1.5329 (15)	C16—C17	1.5235 (16)
C2—H2A	0.9900	C16—H16A	0.9900
C2—H2B	0.9900	C16—H16B	0.9900
C3—C4	1.5278 (16)	C17—C18	1.5305 (14)
C3—H3A	0.9900	C17—H17A	0.9900
C3—H3B	0.9900	C17—H17B	0.9900
C4—C5	1.5255 (16)	C18—H18A	0.9900
C4—H4A	0.9900	C18—H18B	0.9900
C4—H4B	0.9900	C19—C20	1.5230 (14)
C5—C6	1.5274 (15)	C19—C24	1.5262 (14)
C5—H5A	0.9900	C19—H19	1.0000
C5—H5B	0.9900	C20—C21	1.5303 (14)
C6—H6A	0.9900	C20—H20A	0.9900
C6—H6B	0.9900	C20—H20B	0.9900
C7—C8	1.5227 (15)	C21—C22	1.5257 (15)
C7—C12	1.5273 (15)	C21—H21A	0.9900
C7—H7	1.0000	C21—H21B	0.9900
C8—C9	1.5317 (15)	C22—C23	1.5240 (15)
C8—H8A	0.9900	C22—H22A	0.9900
C8—H8B	0.9900	C22—H22B	0.9900
C9—C10	1.5235 (17)	C23—C24	1.5290 (14)
C9—H9A	0.9900	C23—H23A	0.9900
C9—H9B	0.9900	C23—H23B	0.9900
C10—C11	1.5277 (17)	C24—H24A	0.9900
C10—H10A	0.9900	C24—H24B	0.9900
			
C7—N1—C1	117.79 (8)	C7—C12—H12A	109.6
C7—N1—H11	108.0 (9)	C11—C12—H12A	109.6
C1—N1—H11	108.7 (9)	C7—C12—H12B	109.6
C7—N1—H12	107.6 (9)	C11—C12—H12B	109.6
C1—N1—H12	108.4 (9)	H12A—C12—H12B	108.1
H11—N1—H12	105.8 (13)	N2—C13—C18	110.76 (8)
C13—N2—C19	117.74 (8)	N2—C13—C14	107.88 (8)
C13—N2—H21	107.2 (9)	C18—C13—C14	112.09 (8)
C19—N2—H21	107.9 (9)	N2—C13—H13	108.7
C13—N2—H22	107.4 (10)	C18—C13—H13	108.7
C19—N2—H22	107.2 (10)	C14—C13—H13	108.7
H21—N2—H22	109.2 (13)	C13—C14—C15	109.75 (8)
N1—C1—C2	110.71 (8)	C13—C14—H14A	109.7
N1—C1—C6	107.72 (8)	C15—C14—H14A	109.7
C2—C1—C6	111.89 (9)	C13—C14—H14B	109.7
N1—C1—H1	108.8	C15—C14—H14B	109.7
C2—C1—H1	108.8	H14A—C14—H14B	108.2
C6—C1—H1	108.8	C16—C15—C14	110.52 (9)
C1—C2—C3	110.68 (9)	C16—C15—H15A	109.5
C1—C2—H2A	109.5	C14—C15—H15A	109.5
C3—C2—H2A	109.5	C16—C15—H15B	109.5
C1—C2—H2B	109.5	C14—C15—H15B	109.5
C3—C2—H2B	109.5	H15A—C15—H15B	108.1
H2A—C2—H2B	108.1	C17—C16—C15	110.38 (9)
C4—C3—C2	111.76 (9)	C17—C16—H16A	109.6
C4—C3—H3A	109.3	C15—C16—H16A	109.6
C2—C3—H3A	109.3	C17—C16—H16B	109.6
C4—C3—H3B	109.3	C15—C16—H16B	109.6
C2—C3—H3B	109.3	H16A—C16—H16B	108.1
H3A—C3—H3B	107.9	C16—C17—C18	111.81 (9)
C5—C4—C3	110.38 (10)	C16—C17—H17A	109.3
C5—C4—H4A	109.6	C18—C17—H17A	109.3
C3—C4—H4A	109.6	C16—C17—H17B	109.3
C5—C4—H4B	109.6	C18—C17—H17B	109.3
C3—C4—H4B	109.6	H17A—C17—H17B	107.9
H4A—C4—H4B	108.1	C13—C18—C17	110.24 (9)
C6—C5—C4	110.78 (9)	C13—C18—H18A	109.6
C6—C5—H5A	109.5	C17—C18—H18A	109.6
C4—C5—H5A	109.5	C13—C18—H18B	109.6
C6—C5—H5B	109.5	C17—C18—H18B	109.6
C4—C5—H5B	109.5	H18A—C18—H18B	108.1
H5A—C5—H5B	108.1	N2—C19—C20	109.85 (8)
C5—C6—C1	110.93 (9)	N2—C19—C24	107.63 (8)
C5—C6—H6A	109.5	C20—C19—C24	112.26 (8)
C1—C6—H6A	109.5	N2—C19—H19	109.0
C5—C6—H6B	109.5	C20—C19—H19	109.0
C1—C6—H6B	109.5	C24—C19—H19	109.0
H6A—C6—H6B	108.0	C19—C20—C21	110.61 (9)
N1—C7—C8	110.57 (9)	C19—C20—H20A	109.5
N1—C7—C12	108.39 (8)	C21—C20—H20A	109.5
C8—C7—C12	111.61 (9)	C19—C20—H20B	109.5
N1—C7—H7	108.7	C21—C20—H20B	109.5
C8—C7—H7	108.7	H20A—C20—H20B	108.1
C12—C7—H7	108.7	C22—C21—C20	111.33 (9)
C7—C8—C9	110.00 (9)	C22—C21—H21A	109.4
C7—C8—H8A	109.7	C20—C21—H21A	109.4
C9—C8—H8A	109.7	C22—C21—H21B	109.4
C7—C8—H8B	109.7	C20—C21—H21B	109.4
C9—C8—H8B	109.7	H21A—C21—H21B	108.0
H8A—C8—H8B	108.2	C23—C22—C21	110.51 (9)
C10—C9—C8	111.25 (10)	C23—C22—H22A	109.5
C10—C9—H9A	109.4	C21—C22—H22A	109.5
C8—C9—H9A	109.4	C23—C22—H22B	109.5
C10—C9—H9B	109.4	C21—C22—H22B	109.5
C8—C9—H9B	109.4	H22A—C22—H22B	108.1
H9A—C9—H9B	108.0	C22—C23—C24	111.30 (9)
C9—C10—C11	110.86 (10)	C22—C23—H23A	109.4
C9—C10—H10A	109.5	C24—C23—H23A	109.4
C11—C10—H10A	109.5	C22—C23—H23B	109.4
C9—C10—H10B	109.5	C24—C23—H23B	109.4
C11—C10—H10B	109.5	H23A—C23—H23B	108.0
H10A—C10—H10B	108.1	C19—C24—C23	111.28 (9)
C10—C11—C12	110.75 (10)	C19—C24—H24A	109.4
C10—C11—H11A	109.5	C23—C24—H24A	109.4
C12—C11—H11A	109.5	C19—C24—H24B	109.4
C10—C11—H11B	109.5	C23—C24—H24B	109.4
C12—C11—H11B	109.5	H24A—C24—H24B	108.0
H11A—C11—H11B	108.1	N3—C25—S1	178.87 (10)
C7—C12—C11	110.43 (9)	N4—C26—S2	179.22 (10)
			
C7—N1—C1—C2	−63.27 (11)	C19—N2—C13—C18	−58.85 (11)
C7—N1—C1—C6	174.10 (9)	C19—N2—C13—C14	178.14 (8)
N1—C1—C2—C3	−174.30 (9)	N2—C13—C14—C15	179.34 (8)
C6—C1—C2—C3	−54.13 (12)	C18—C13—C14—C15	57.15 (11)
C1—C2—C3—C4	54.77 (13)	C13—C14—C15—C16	−57.84 (11)
C2—C3—C4—C5	−56.51 (14)	C14—C15—C16—C17	57.81 (12)
C3—C4—C5—C6	57.23 (13)	C15—C16—C17—C18	−56.46 (12)
C4—C5—C6—C1	−56.84 (13)	N2—C13—C18—C17	−175.91 (8)
N1—C1—C6—C5	177.42 (9)	C14—C13—C18—C17	−55.37 (11)
C2—C1—C6—C5	55.53 (12)	C16—C17—C18—C13	54.81 (12)
C1—N1—C7—C8	−65.18 (12)	C13—N2—C19—C20	−54.20 (11)
C1—N1—C7—C12	172.18 (9)	C13—N2—C19—C24	−176.70 (8)
N1—C7—C8—C9	−177.40 (9)	N2—C19—C20—C21	−174.01 (8)
C12—C7—C8—C9	−56.65 (13)	C24—C19—C20—C21	−54.29 (12)
C7—C8—C9—C10	56.46 (14)	C19—C20—C21—C22	55.96 (12)
C8—C9—C10—C11	−56.77 (14)	C20—C21—C22—C23	−57.26 (12)
C9—C10—C11—C12	56.41 (15)	C21—C22—C23—C24	56.46 (13)
N1—C7—C12—C11	178.84 (9)	N2—C19—C24—C23	174.89 (8)
C8—C7—C12—C11	56.83 (12)	C20—C19—C24—C23	53.89 (12)
C10—C11—C12—C7	−56.15 (14)	C22—C23—C24—C19	−54.72 (13)

### Hydrogen-bond geometry (Å, °)

**Table:**

*D*—H···*A*	*D*—H	H···*A*	*D*···*A*	*D*—H···*A*
N1—H11···N3	0.887 (15)	2.091 (15)	2.9696 (13)	170.6 (13)
N1—H12···N4^i^	0.960 (16)	1.900 (16)	2.8539 (13)	172.1 (13)
N2—H21···N3	0.911 (15)	2.068 (15)	2.9650 (12)	168.1 (13)
N2—H22···S2	0.895 (16)	2.475 (16)	3.3544 (9)	167.4 (13)

Symmetry codes: (i) *x*, *y*, *z*−1.
